# Thiosulfate leaching in carbonaceous gold-bearing ores in Ethiopia

**DOI:** 10.1038/s41598-024-69646-3

**Published:** 2024-10-03

**Authors:** Kaleb Jia Chaka, Steven M. Rupprecht

**Affiliations:** 1 Unity University, Addis Ababa, Ethiopia; 2 South African Institute of Mining and Metallurgy (SAIMM), Johannesburg, South Africa

**Keywords:** Gold leaching, Carbonaceous gold-bearing ores, Thiosulfate, Biogeochemistry, Environmental sciences, Solid Earth sciences, Chemistry, Engineering

## Abstract

One of the main unit operations in metallurgical processing plant designs is gold leaching. The traditional cyanidation process was chosen and is currently in widespread use. However, the mining sector is looking for lixiviants other than cyanide. Cyanide’s effects on the environment have made it difficult for humans and other biotic creatures to survive. There is now research to discover a substitute for this cyanide. It is currently argued that thiosulfate is a preferable substitute for cyanide. The effectiveness of thiosulfate as a leaching agent in carbonaceous gold-bearing ores in Ethiopia is discussed in this paper compared to cyanide. The study has looked into the advantages of employing thiosulfate over cyanide from a technical, and economic standpoint. The leaching effects of both lixiviants on carbonaceous gold-bearing ores extracted from MIDROC Legadembi Open Pit mine in Southern Ethiopia, Oromia region, were examined in a laboratory experiment. After 48 h of leaching, it was discovered that thiosulfate has a better and quicker recovery of 91.54% over 61.70% of cyanide recovery. Tables and graphs are used to demonstrate thiosulfate’s technical advantage over cyanide. As a result, this paper provides evidence regarding the Legadembi gold mine in Ethiopia’s amenability to thiosulfate leaching on carbonaceous gold-bearing ores. A further research perspective is also sought for thiosulfate leaching in other refractory ores.

## Introduction

Over the past 20 years, there has been an increase in research into alternative lixiviates such as the halogens (Iodine, Bromine, and Chlorine), ammonia, thiocyanate, thiourea, thiosulfate, polysulfides, sulfite, and diethylamine for the treatment of problematic ores^1^. Increased environmental pressure to ban or limit the use of cyanide in metallurgical plants throughout the world is a prime motivator for research into alternatives to cyanide. Some of these alternatives offer a safer and environmentally sound extraction method and, for some ores, these lixiviants can also increase the recovery of gold^2,8^. Except for chlorine, little commercial use has been made of alternative lixiviants, but several have been tested in pilot-scale plants. The most favoured current alternative to cyanide for the treatment of problematic ores is thiosulfate^3,9^.

Before these lixiviants, the international mining community used highly toxic sodium cyanide (NaCN) to extract gold and other precious metals through milling high-grade ores and heap leaching of low-grade ores^10^. The process to concentrate gold using cyanide was developed in Scotland in 1887 and was used almost immediately in the Witwatersrand gold fields of the Republic of South Africa. Heap leaching with cyanide was proposed by the U.S. Bureau of Mines in 1969 as a means of extracting gold from low-grade ores. The gold industry adopted the technique in the 1970s, soon making heap leaching the dominant technology in gold extraction^4^.

The process of gold dissolution in cyanide (and consequently the extraction of gold from its ores by cyanide) involves heterogeneous reactions at the solid–liquid interfaces. Hence, the following sequential steps may be assumed to lead to the dissolution of gold (from its ores) by cyanide:

I. Absorption of oxygen in solution,
II. Transport of dissolved cyanide and oxygen to the solid–liquid interface,
III. Adsorption of the reactants (CN^-^ and O_2_) on the solid surface,
IV. Electrochemical reaction,
V. Adsorption of the soluble gold-cyanide complexes and other reaction products from the solid surface,
VI. Transport of the adsorbed products into the bulk of solutions.

The dissolution of gold is an oxidation–reduction process in which cyanide ions form a strong complex with Au+ ions. The stable complex ion is (Au (CN)^2−^). The overall reaction, where oxygen is reduced and hydrogen peroxide is formed to be the oxidizing agent in the second step, is presented below:

1 $$2\text{Au}+2{\text{CN}}^{-}+{\text{O}}_{2}+{2\text{H}}_{2}\text{O}=2[\text{Au}{\left(\text{CN}\right)2]}^{-}+{\text{H}}_{2}{\text{O}}_{2}+20{\text{H}}^{-1}$$

2 $$2\text{Au}+4{\text{CN}}^{-}+{\text{H}}_{2}{\text{O}}_{2}=2[\text{Au}{\left(\text{CN}\right)2]}^{-}+20{\text{H}}^{-}$$

3 $$2\text{Au}+4{\text{CN}}^{-}+{\text{O}}_{2}+2{\text{H}}_{2}\text{O}=2[\text{Au}{\left(\text{CN}\right)2]}^{-}+40{\text{H}}^{-}$$

However, in recent years, thiosulfate has been considered the most attractive alternative to cyanide for leaching gold, with many investigations taking place worldwide. This is primarily based on its low toxicity and potential use on ‘preg-robbing’ carbonaceous ores that conventional cyanidation cannot readily treat. It has been known for over a hundred years that gold can be leached with thiosulfate. Thiosulfate was the main competitor to cyanide in the 1880s when there was an increase in research to improve gold leaching and recovery from the existing gravity and mercury amalgamation processes.

Thiosulfate is a structural analogue of sulfate, replacing one oxygen atom with a sulfur atom. This gave thiosulfate its former name of hyposulfite^5,11^. The structure S–SO_3_^–2^ is dominated by the sulfur atom, which gives thiosulfate its reducing properties, strong complexing tendencies, and sulfide-forming capabilities^5^. The proposed mechanism for the dissolution of gold in ammoniacal thiosulfate solutions is more complex than portrayed here. It has been suggested that gold is oxidised by copper (II), and the gold diamine complex is subsequently converted to the gold thiosulfate complex as shown below^6,12^:

4 $$\text{Au}+\text{Cu}{\left({\text{NH}}_{3}\right)}_{4}^{+2}+2{\text{S}}_{2}{\text{O}}_{3}^{-2}=\text{Au}{\left({\text{NH}}_{3}\right)}_{2}^{+}+\text{Cu}{\left({\text{S}}_{2}{\text{O}}_{3}\right)}_{2}^{-3}+2{\text{NH}}_{3}$$

5 $$\text{Au}{\left({\text{NH}}_{3}\right)}_{2}^{+}+ 2{\text{S}}_{2}{\text{O}}_{3}^{-2}=\text{ Au}{\left({\text{S}}_{2}{\text{O}}_{3}\right)}_{2}^{-3}+ 2{\text{NH}}_{3}$$

In recent years, thiosulfate leaching system, especially the traditional copper–ammonia–thiosulfate system has been studied extensively. Many researchers considered thiosulfate is the most promising alternative to cyanidation. However, with many years of process development, there are still some problems and challenges that the thiosulfate-based leaching systems must face to. These include (but not limited) the high consumption of thiosulfate, the passivation of gold surface, the potential hazard of ammonia, and more critically that, the gold leaching kinetics in thiosulfate solutions at ambient temperature is dramatically slow though the gold extraction rate is also readily affected by other operating conditions such as pH and dissolved oxygen, and the type of gold ores^7^.

This paper aims to examine the economic implications of utilising thiosulfate for gold leaching in ores that are carbonaceous (preg-robbing) and contain gold. The technical feasibility has been demonstrated in connection with an improved leaching recovery or efficiency compared to the conventional cyanidation process. As there are few publications on the topic, this paper will add to the current body of knowledge on utilising thiosulfate for gold leaching in carbonaceous ores. The authors hope this study will provide the reader with information about the economic, and technical aspects of employing thiosulfate lixiviants instead of cyanide leaching, specifically in Ethiopia’s gold ore. The findings will be particularly important to Ethiopia’s gold metallurgical environment and future thiosulfate leaching research undertaken by other academics.

## Study methodology

First, a comprehensive analysis of the characteristics of carbonaceous ores and how they could be processed using conventional cyanidation methods was conducted. Numerous books, journals, and research papers were examined for this purpose. Next, research was done on the use and technical feasibility of thiosulfate as a gold-leaching agent. Studies have also been conducted on the effects of associated reagents used in leaching. In light of this, the authors have attempted to validate their findings by performing laboratory experiments on the leaching process. This has aided in establishing the effect of some of the parameters under study.

Lastly, the economics of employing thiosulfate leaching over conventional cyanidation methods for carbonaceous gold-bearing ores have been examined. The findings of the technical inquiry are then presented, together with the economic conclusions.

Carbonaceous refractory gold ores contain organic carbon and inorganic carbon from black (or carbonaceous) and sedimentary rock series. The carbonaceous gold ores are refractory because dissolved aurocyanide complex is robbed by adsorption of carbonaceous matter, and this phenomenon is termed preg-robbing. The most important carbonaceous preg-robbers are elemental carbon and organic carbon, and more than half organic carbon is humic acid. The preg-robbing behavior of elemental carbon is similar with activated carbon, while the humic acid forms complex with gold by functional groups. Generally, if the content of organic carbon is above 0.2%, it will seriously disturb gold extraction cyanidation.

### Experimental method

In a laboratory, the amenability of carbonaceous gold-bearing ores to thiosulfate leaching was tested. The test aimed to demonstrate the technical benefits of thiosulfate leaching over conventional cyanidation.

MIDROC Legadembi’s operating conditions provided for the ideal cyanidation settings and optimal leaching conditions for thiosulfate lixiviants were taken from literatures. Broken ore from the Legadembi Open Pit gold mine was obtained for testing from two locations on the North-East Footwall side, which contained 30 kg carbonaceous gold bearing ores with grades greater than 1 g/t. The Legadembi mine is known for its sulfide gold mineralized vein type deposit. Pyrite (FeS2), arsenopyrite (FeAsS), chalcopyrite (CuFeS_2_), galena (PbS) and sphalerite (ZnS) are common sulfide minerals found in this vein.

The following is a presentation of the test findings and laboratory experiments.

The samples were oven-dried for over 20 h to evaporate the surface moisture of the ore, then ground to P80 (80% passing) of 75 µm in a pulverizer for five minutes.

To create a typical sample ore, the two ore samples were mixed to a 1:1 ratio; then the samples were mixed in a tumbler machine for 30 min to guarantee that they were distributed evenly.

### Leaching experiment

Before the leaching test, a sample of the ore was sent to the Analytical laboratory for gold analysis by fire assay; and it was found through triplet sample analysis that the ore sample had a gold grade of 1.25 g/t. Please see Table 1 below:

**Table 1:** Sample gold assay triplet analysis.

Sample no	Grade (g/t)	Average grade (g/t)
Sample no. 1	1.34	
Sample no. 2	1.19	1.25
Sample no. 3	1.22	

The combined ore sample was subjected to a series of bottle leach tests to ascertain the reagent concentration needed to recover the largest amount of gold. All the regents were dissolved in a beaker, and quick lime (CaO) was used to raise the pH from 10.0 to 11.5. The pH of the solution was measured using a pH meter. While Tests 3 and 4 had one parameter changed from the base case scenarios and were also conducted in parallel, Tests 1 and 2 were the base case scenarios. The tests that were run and the parameters examined for this study are briefly shown in the following summary.

### Laboratory test summary

*Activity* Thiosulfate leaching Vs. Cyanide leaching in Carbonaceous Gold Bearing Ores.

*Duration* April 12th–22nd 2016.

*Venue*: MIDROC Gold Mine Legadembi Laboratory Services.

*Laboratory test conditions*

- Keep the pulp density constant at 50% solid (pulp density) and vary the concentration of the lixiviants
- Keep the pH range 10.0–11.5
- Keep the leaching process going for 48 h. residence time (or leaching time).

General test conditions, which are common for both lixiviants, such as % solid, pH, residence time, etc., were kept constant as ground for comparing the two leaching agents. The initial solution strength will vary, and the focus will be on the free cyanide in the solution to see the preg-robbing effect and to make up for the lost cyanide for assisting the leach. See Table 2 below for the summary of laboratory test conditions.

**Table 2:** Laboratory test conditions.

No	Experimental conditions	First run	Second run
1	Head grade	1.25 g/t	1.25 g/t
2	Sodium cyanide (NaCN) strength	0.5 g/l	0.25 g/l
3	Sodium thiosulfate (Na2S2O3) strength	9 g/l	8 g/l
4	Ammonia (NH3)	13.6 g/l	13.6 g/l
5	Copper sulfate (CuSO4)	0.8 g/l	0.8 g/l
6	Sample size	2 kg	2 kg
7	Pulp density	50% Solid	50% Solid
8	pH	10.0	11.5
9	Leaching time	48 h	48 h
10	Temperature	20–25 °C (Room temp.)	20–25 °C (Room temp.)

*Procedure*

1. Take a sample and crush it to a size of − 10 mm with a jaw crusher
2. Take the crushed sample and crush it again to a size of − 1 mm with a Boyd crusher (for 5 min)
3. Then pulverize (grind) the crushed material to a size of − 75 microns to liberate the metal (Au) inside the ore
4. Then, with a manual splitter, obtain a representative sample of about 2 kg for the tests (four samples)
5. Prepare the reagents to be added with the appropriate concentration strength (NB: all reagent concentration are indicated for the parent chemical formula as depicted on the table below)
6. Prepare a 50% solid sample by adding two litres of water to the prepared 2 kg samples
7. Adjust the pH of the pulp to a range between 10.0 and 11.5 by adding lime (CaO) accordingly
8. Finally, add the reagents to their respective leach bottles into the prepared pulp and start agitating the mix to 200 rpm to promote homogeneity and increase the kinetics of the reaction, thereby increasing the leaching process
9. Take leaching samples to check the progress of the dissolution of gold with the proposed lixiviants

## Result and discussion

This paper has attempted to demonstrate the technical superiority of thiosulfate over cyanide through laboratory testing. Table 3 provide the results of the two test runs, considering the variations in general leach circumstances. A conclusion has been reached by comparing the chemical properties of cyanide and thiosulfate lixiviants in order to illustrate the economic aspects of leaching.

**Table 3:** First run laboratory results.

Test-1	Sodium thiosulfate (9 g/l)			
Time (hr.)	Au in solution (g/t)	Percent recovery (%)	pH	Free TS (ppm)
0	0.00	0.00	10.0	ND
2	0.32	25.64	11.18	ND
4	0.40	32.05	10.90	ND
8	0.52	41.67	10.62	ND
24	0.71	56.89	10.07	ND
30	0.74	59.29	9.79	ND
48	1.13	90.54	10.24	ND

Test-2	Sodium cyanide (0.5 g/l)			
Time (hr.)	Au in solution (g/t)	Percent recovery (%)	pH	Free CN^-^ (ppm)
0	0.00	0.00	10.0	500
2	0.26	20.83	10.26	460
4	0.35	28.04	10.24	450
8	0.51	40.87	9.97	350
24	0.63	50.48	9.86	300
30	0.72	57.69	10.65	285
48	0.77	61.70	10.24	200

### First run

For the two leaching agents mentioned above, comparable test circumstances were employed. Therefore, as Table 3 illustrates, thiosulfate exhibits more technical superiority over cyanide, producing a gold recovery in a solution of approximately 90.54% compared to cyanide’s 61.70% after 48 h of leaching. This suggests that in carbonaceous preg-robbing gold-bearing ores, thiosulfate has a far superior leaching recovery than cyanide.

### Second run

The outcome of the fourth test as depicted in Table 4 demonstrates the preg-robbing impact of carbonaceous gold-bearing ores as leaching progresses and eliminates the free cyanide in the solution. Due to the nature of the ore, which absorbs free cyanide in solution to form other undesirable cyanide complexes, there is a significant loss of free cyanide in solution, as demonstrated by the cyanide leaching, which shows 35 ppm of free cyanide in solution at the beginning of the leaching process. Nonetheless, it is essential to note that for the gold dissolution reaction to occur, the solution must include at least 100–150 ppm of free cyanide. To make up for the lost cyanide in the solution, 0.15 g/l of free cyanide was added to the leach solution on the fourth and twenty-fourth hours. This has increased the processing costs of the cyanidation leaching for carbonaceous gold-bearing ores and have a negative economic impact due to overdosing on cyanide dosage.

**Table 4:** Second run laboratory results.

Test-3	Sodium thiosulfate (8 g/l)			
Time (hr.)	Au in solution (g/t)	Percent recovery (%)	pH	Free TS (ppm)
0	0.00	0.00	11.50	ND
2	0.41	32.85	11.67	ND
4	0.46	36.86	11.61	ND
8	0.52	41.67	11.44	ND
24	0.74	59.29	11.59	ND
30	0.78	62.50	11.51	ND
48	0.83	66.51	11.18	ND

Test-4	Sodium cyanide (0.25 g/l)			
Time (hr.)	Au in solution (gm/ton)	Percent recovery (%)	pH	Free CN^-^ (ppm)
0	0.00	0.00	11.92	250
2	0.22	17.63	11.69	150
4	0.30	24.04	11.60	35 + 150
8	0.37	29.65	11.42	150
24	0.33	26.44	11.72	100 + 150
30	0.45	36.06	11.35	200
48	0.48	38.46	11.21	150

The result implies a faster leach recovery by thiosulfate, which also demonstrates a higher leaching recovery of 66.51% of thiosulfate over 26.44% cyanide leach in 24 h of residence time. The results presented here solely demonstrate thiosulfate’s technical advantage over cyanide. However, greater recovery may be obtained if conditions could be improved to a better state with a thorough study to be conducted by adjusting reagent concentrations and other leach conditions.

The result also shows a superior leaching recovery of 66.51% of thiosulfate over 26.44% cyanide leach in 24 h. of residence time, implying a faster leach recovery by thiosulfate. The figures here only indicate the technical superiority of thiosulfate over cyanide. But, if conditions could be optimized to a better state with rigorous research to be undertaken by varying reagent concentrations and other conditions for leach, a much better recovery and sound figures could be obtained. The preg-robbing effect, in which the leached gold in solution is stolen by the free carbon and other hydrocarbons from the carbonaceous ore, is further demonstrated by the lower percent recoveries and changes in recovery for cyanide in Test 4. This resulted in a significant drop of gold in the solution.

The other thing the authors noticed in the test results was the status of the solution’s alkalinity for the best thiosulfate recovery. Test-1, conditioned at pH 10.0, produced a higher recovery of around 90.54%, while Test 3, conditioned at pH 11.5, produced a solution recovery of 62.5%. This study unequivocally demonstrated that the lower alkaline bound, or pH range of 9–10.5, yields a better and faster recovery of gold in thiosulfate solution. This pH range allows for the best possible recovery of gold in thiosulfate solutions.

Finally, the gold left in the solid as residue after being fire assayed on finalizing the leaching process was examined in the laboratory, and the result is shown in Table 5:

**Table 5:** Gold grade in the solid residue.

Test no	Solid grade (Residue, g/t)	Au in solution after 48 h (g/t)	Head grade (g/t)
Test-1 (Sodium thiosulfate)	0.12	1.13	
Test-2 (Sodium Cyanide)	0.50	0.77	1.25
Test-3 (Sodium thiosulfate)	0.45	0.78	
Test-4 (Sodium Cyanide)	0.68	0.48	

Head grade = 1.25 g/t.

Residue yield (%) = (Final Grade/Initial Grade)*100.

Taking the average of the two tests for the Thiosulfate leaching solid residue grade of 0.285 g/t, the residue yield results in 22.8%. This indicates about 22.8% of the gold is lost undissolved in the leaching process if the Thiosulfate leaching process remains un-optimized with rigorous test works to counter this loss.

With the same calculation and taking the average solid residue of 0.59 g/t for the two test runs of cyanide leaching process, the residue yield results in 47.2%. This clearly shows almost half of the gold in the preg-robbing gold minerals are lost undissolved in the leaching process. Hence, the comparison between these two lixiviants based on residual loss vividly represents the technical superiority of thiosulfate lixiviants over conventional cyanide leaching processes as reflected and discussed on results in liquid data in previous section.

### Economic result

This section compares the costs of cyanidation with thiosulfate leaching of gold ores. In addition to the primary operational expenses incurred during the conventional cyanidation process of detoxifying the cyanide from the tailing management facility, expenditures related to direct chemical consumption are also utilised for both lixiviants. Table 6 lists the costs along with the process for analyzing them.

**Table 6:** Leaching reagent consumption and costs.

Chemical name	Unit	Unit price (Ethiopian Currency, Birr)	Specific consumption (Kg/t)	Amount (Birr/t)
Sodium cyanide	Kg	81.69	0.80	65.35
Sodium thiosulfate	Kg	16.60	9.00	149.40
Ammonia	Kg	9.00	13.60	122.40
Copper sulfate	Kg	44.00	0.80	35.20
Hypochlorite	Kg	79.64	0.02	1.59

The Legadembi metallurgy plant at MIDROC Gold Mine provided the following reagent cost, which is based on the current market pricing in Ethiopian Birr.

Taking a particular case for MIDROC Legadembi mine, a facility which processes 1,100,000 tons of gold ore annually with the conventional cyanidation method to estimate operating costs; the detox plant uses a minimum of 20 tonnes of hypochlorite per annum for treating cyanide in tailing solution and taking an average power consumption charge of 0.60 Birr/KWh by EEPCO. Table 7 provides information about operating cost analysis for the detox process, with cost details depicted on Tables 8 and 9.

**Table 7:** Detoxification operating cost analysis.

Item no	Major items	Costs per annum (Birr)
1	Detox plant operating items (*Details: *Table 8)	–
1.1	Spare part replacements	950,000.00
1.2	Running items and Utilities	446,266.80
2	Tailing dam operating items (*Details: *Table 9*)*	–
2.1	Spare part replacements	2,610,000.00
2.2	Running items and Utilities	549,564.00
3	Supervisory fee for two Units (One person working for 8h/day with monthly salary of 10,000BIRR)	120,000.00
	Grand Total	4,621,080.80
	Annual budgeted plant processing capacity	1.1 million tons
	Unit cost for detoxification of cyanide tailings (Birr/t)	4.20

**Table 8:** Detox plant operating items.

Item no	Spare part replacements	Costs per annum (Birr)
1	Electrical parts (breakers, contactors, cables etc.)	200,000.00
2	Pump parts	300,000.00
3	Pipes and Valve replacements	250,000.00
4	Tank spare parts (gearbox and others)	200,000.00
	Total	950,000.00

Item no	Running items and utilities	Costs per annum (Birr)
1	Consumables for Cyanide titration & pH determination	54,750.00
2	Power consumption for:	
2.1	Mixing tank agitator at 11KW capacity working for 24 h per day	57,816.00
2.2	Transfer pump at 11KW capacity working for 24 h per month	1900.80
2.3	Dozing pump at 10KW capacity working for 20 h per day	43,800.00
3	Man power (four operators with 6000 Birr monthly salary each)	288,000.00
	Total	446,266.80

**Table 9:** Tailing dam operating items.

Item no	Spare part replacements	Costs per annum (Birr)
1	Electrical parts (breakers, contactors, cables etc.)	100,000.00
2	Pump parts	1,200,000.00
3	Return pipe and fitting replacements	1,100,000.00
4	Hose and Valve parts	150,000.00
5	Bearing replacements	60,000.00
	Total	2,610,000.00

Item no	Running items and utilities	Costs per annum (Birr)
1	Consumables for cyanide titration & pH determination	54,750.00
2	Power consumption for:	
2.1	Two return pumps at 11KW capacity working for 23 h per day	110,814.00
3	Man power (Eight operators with 4000 Birr monthly salary each)	384,000.00
	Total	549,564.00

Pre-treatment of ores is mandatory for refractory ores before leaching with cyanide. Thus, roasting of ores as pre-treatment method has been adopted for this economic analysis. The roasting cost for carbonaceous ores here has been estimated with taking a particular case of Derba Cement’s clinker production kiln (Table 10), a major cement producing company in Ethiopia.

- The Derba kiln uses around 735 KCAL of energy to produce 1 kg of clinker
- The cement plant mostly uses lignite coals as energy source with caloric value of around 2310 KCAL/Kg
- Lignite coal types are sold with 938.40 Birr per tons at international market including 20% for overhead cost

**Table 10:** Roasting cost estimation.

Pre-treatment (roasting cost)		Economic output
Kiln coal requirement	0.32 kg/Kg of clinker	300.80 Birr/t of clinker
Lignite unit price	0.94 Birr/Kg	

The cement plant kiln for clinker production could here be taken as equivalent to a kiln used for roasting of refractory ores to produce pre-treated ores for cyanide leaching for estimating roasting cost; which resulted in 300.80 BIRR/t of ore pre-treated.

In summary, the above cost analysis gives the following summarised costs for both lixiviants as shown in Table 11:

**Table 11:** Leaching economics result.

Leaching method	Total unit cost (Birr/t)
Cyanide leaching	371.94
Thiosulfate leaching	307.00

The result demonstrates that thiosulfate leaching of gold also benefits the economy. By improving the thiosulfate leach process for refractory ores, which would probably reduce the requirement of higher concentrations of reagents in the leach system, this economic benefit of thiosulfate might be further utilised. The increased cost of the conventional cyanidation process demonstrates why such types of refractory ores are not treated using this method. Thiosulfate recovery offers a considerable advantage over cyanide, as can be seen from the test results. This means that there will be an increase in revenue as the recovery is increased by utilising thiosulfate. Furthermore, quantifying the non-environmental benefits of thiosulfate vs cyanide in monetary terms would undoubtedly increase the thiosulfate leach system’s economic advantage for carbonaceous ores.

Thiosulfate is considered as the most promising alternative reagent to cyanide, especially, in dealing with copper-bearing or carbonaceous gold ores when using cyanide, or cyanide consumption is high. However, the thiosulfate based gold leaching processes are still far away from large scale application because the overall gold recovery is generally lower and reagent consumption is higher compared to cyanidation. The use of the copper–ammonia–thiosulfate leaching system and its modifications has been studied extensively, both in theory and process development^7^.

In addition, the presence of ammonia in thiosulfate leaching system will produce a large amount of wastewater and tailings containing ammonia or ammonium, which may cause great damages to the surrounding environment^7^.

## Conclusion and recommendation

The results of this paper support the hypothesis that thiosulfate will improve and speed up the recovery when leaching carbonaceous gold-bearing ores. The following observations and conclusions are made.

The current study indicates that thiosulfate, at a concentration of 9 g/l, has a higher recovery (90.54%) in carbonaceous gold-bearing ores than cyanide (61.70%). If more research were conducted on the general test circumstances, altering the crucial factors such as pulp density (% solid), reagent concentration, pH, and so on, the results could be enhanced even more.

The results also indicate that, with a minimum residence time of 24 h, thiosulfate could dissolve gold in carbonaceous gold-bearing ores more quickly than other chemicals, with a 66.51% recovery rate. This phenomenon might be further exploited to reach the 73% recovery within 4 h of residence time.

Because the primary chemical agents in the leaching process (sodium thiosulfate and ammonia solution) are popular fertilizers, using mine tailings in agricultural applications is also possible in countries where environmental regulations are very stringent.

Due to cyanide’s inefficiency with some refractory ore types, the decrease in free milling ore also increases the need for thiosulfate leaching. Thiosulfate lixiviants can be used for various purposes in these and related illnesses.

To ascertain the precise circumstances in which thiosulfate could benefit the mining industry, a thorough investigation of the electrochemistry of the leach process and concepts related to the reduction potential and Gibbs free energy are necessary and should be studied seriously.

## Data Availability

We, the authors, consent that the datasets used and/or analysed during the current study are available from the corresponding author on reasonable request.
